# Crosstalk of four kinds of cell deaths defines subtypes of cutaneous melanoma for precise immunotherapy and chemotherapy

**DOI:** 10.3389/fimmu.2022.998454

**Published:** 2022-11-30

**Authors:** Qi Wan, Ran Wei, Xin Wei, Ying-ping Deng

**Affiliations:** Department of Ophthalmology, West China Hospital, Sichuan University, Chengdu, Sichuan, China

**Keywords:** cell deaths, cutaneous melanoma, chemotherapy, immunotherapy, subtype

## Abstract

**Background:**

Cell death patterns can give therapeutic and biological clues that facilitate the development of individualized treatments for this lethal form of skin cancer.

**Methods:**

We employed unsupervised clustering to establish robust classifications based on the four kinds of cell death-associated gene expression of 462 melanoma patients in the Cancer Genome Atlas (TCGA) and tested their reproducibility in two independent melanoma cohorts of 558 patients. We then used dimensionality reduction of graph learning to display the different characteristics of cell death patterns and immune microenvironments.

**Results:**

We examined 570 cell death-associated gene expression data of melanoma patients for exploration, independent verification, and comprehensive classification of five reproducible melanoma subtypes (CS1 to CS5) with different genomic and clinical features. Patients in death-inactive subtypes (CS1, CS2, and CS5) had the least immune and stromal cell infiltration, and their prognosis was the poorest. A death-active subtype (CS4), on the other hand, had the highest infiltrated immune and stromal cells and elevated immune-checkpoints. As a result, these patients had the highest response to immunotherapy and the best prognosis. An additional subtype (CS3) had more diversified cell death and immune characteristics with moderate prognoses. Based on graph learning, we successfully divided the CS3 subtype into two subgroups (group A and group B) with distinct survival outcomes and immune features. Finally, we identified eight potential chemical drugs that were specifically targeted for the therapy of melanoma subtypes.

**Conclusions:**

This research defines the intrinsic subtypes of melanoma based on the crosstalk of four kinds of cell deaths, which affords a blueprint for clinical strategies and guiding precise immunotherapy and chemotherapy for melanoma patients.

## 1 Introduction

Cutaneous melanoma is the most lethal type of skin cancer and is caused by the malignant alteration of melanocytes (1). The prevalence of melanoma has steadily risen during the last few decades (2). Therapy procedures have considerably improved in recent years as a result of a better knowledge of the major oncogenes and signaling pathways involved in its development and progression—for instance, molecularly targeted therapies such as the inhibitors of BRAF and MEK and immune-checkpoint blockade like anti-PD1 and CTLA-4 treatments have now significantly improved survival in these melanoma patients (3, 4). However, responses to these therapies are still inconsistent. Many individuals do not benefit at all or relapse after a brief time of remission. Therefore, future results in these melanomas will be dependent on the discovery of new treatment targets and strategies to improve the present targets and immunotherapies.

Cell death is significant in the formation of the organism and sustains homeostasis to avoid disease development (5). Melanoma growth, like other types of cancer, is influenced by several cell death mechanisms and tumor microenvironments (6). The diverse microenvironments of cell death processes influence the immune response owing to the changes in tumor cell death and immune, and stromal cell activities. Thus, treatment efforts should focus on various cell death processes. Cell death is traditionally classified into programmed and non-programmed types depending on the modulation of the underlying processes (7). There are three classical types of programmed cell death (PCD) including apoptosis, necroptosis, and pyroptosis. Apoptosis belongs to non-lytic cell death, while necroptosis and pyroptosis mainly refer to lytic cell death (8, 9). These types of cell death cause intracellular component leakage, containing damage-associated molecular pattern molecules, which trigger a significant inflammatory response, also defined as inflammatory death (10). Furthermore, ferroptosis is a novel cell death based on iron regulation, which belongs to PCD but has unique biochemical and morphological variations from other PCDs such as pyroptosis and necroptosis (11, 12). Cell death has been found to influence the development of a variety of chronic illnesses—for example, dysfunction of the apoptosis pathway has a significant role in resistance to traditional anticancer medicines like targeted therapy, chemotherapy, and radiotherapy (13). Ferroptosis dysregulation is connected to carcinogenesis and has been proven to link the formation of melanoma, breast, gastric, and lymphoma (14). In addition, several research published in the last 5 years have revealed that tumor cells undergoing necroptosis, pyroptosis, and ferroptosis can induce a powerful anti-cancer immunity in *in vitro* and *in vivo* conditions, and their efficacy can be cohesively enhanced by checkpoint blockades, even in cancers with immunotherapy resistance (15–17).

Melanoma is one of the most diverse cancers, with varying degrees of aggressiveness among subtypes that necessitate different treatment strategies. Although stimulation of a specific form of cell death has progressively developed as a new strategy treatment for cancers, most cancers have a built-in resistance to a certain kind of cell death. Therefore, the crosstalk of four kinds of cell deaths (“apoptosis”, “ferroptosis”, “necroptosis”, and “pyroptosis”) has been discussed systematically in this research. Based on cell death-related gene signatures, melanoma patients were successfully classified into distinct subtypes with different clinical characteristics, molecular features, and responses to immunotherapy and chemotherapy.

## 2 Materials and methods

### 2.1 Melanoma collection and process

Publicly accessed databases like Gene-Expression Omnibus (GEO) and the Cancer Genome Atlas (TCGA) were used to retrieve the RNA-seq of melanoma data as well as corresponding clinical annotations. Following data processing steps such as converting to TPMs format, removing low expression values or missing values, and log2-transformation, three melanoma cohorts including the cutaneous melanoma of TCGA data (TCGA_SKCM), meta-GEO data, and meta-immune response data were selected for subsequent analysis. The TCGA_SKCM cohort was used as a training dataset for melanoma classification. Meanwhile, meta-GEO and meta-immune response data were regarded as testing datasets to prove the accuracy of classification. The meta-GEO cohort consisted of four GEO melanoma datasets including GSE19234, GSE53118, GSE54467, and GSE65904. Moreover, the meta-immune response cohort consists of the respective study of Hugo et al. (GSE78220) (18), Riaz et al. (GSE91061) (19), and Van Allen et al. (20), which has detailed information about clinical immunotherapy response. The “ComBat” algorithm of the sva Package was applied to merge several individual studies into the large meta-cohort. This method can reduce batch effects produced by distinct platforms and technological biases.

### 2.2 Four kinds of cell death-related gene

The Molecular Signatures Database in the Gene Set Enrichment Analysis (GSEA) website (https://www.gsea-msigdb.org) and previous literature in the PubMed database were used to compile lists of cell death-related genes. The search keywords include “apoptosis”, “ferroptosis”, “necroptosis”, “pyroptosis”, and “*Homo sapiens*”. After removing the overlapped genes, the list gene sets eventually included the apoptosis-related gene set (*n* = 247 unique genes), ferroptosis-related gene set (*n* = 206 unique genes), necroptosis-related gene set (*n* = 65 unique genes), and pyroptosis-related gene set (*n* = 52 unique genes) (**Supplementary Table S1**).

### 2.3 Clustering of four cell death expression patterns

In order to conduct systematic clustering, the transcriptome data of TCGA_SKCM were obtained to construct four cell death-related gene matrixes with rows corresponding to features and columns corresponding to common samples. Next, unsupervised clustering was applied to discover the potentially significant subtype of melanoma. We employed the “MOVICS” package to perform integrative classification and illustration for cancer subtyping research. The “MOVICS” package afforded two main cluster algorithms (gap statistics and clustering prediction index) to estimate the most possible number of clusters in a partition clustering (21). Therefore, we calculated the gap statistics and clustering prediction index (CPI) to find the best clustering number. As a result, 10 state-of-the-art integrative clustering approaches (CIMLR, iClusterBayes, COCA, ConsensusClustering, MoCluster, NEMO, IntNMF, LRAcluster, PINSPlus, and SNF) were independently performed to systematically cluster the TCGA_SKCM cohort. To boost clustering resilience, we adopted the notion of a consensus ensemble for subsequently classified results produced from diverse methods.

### 2.4 Tumor microenvironment calculation

Two methods for decoding microenvironment cells (ESTIMATE and Stemness Index Workflow) were adjusted to create a compendium of gene lists connected to particular microenvironment cells. The “ESTIMATE” approach was applied to estimate the infiltrated immune cells, stromal cells, and tumor purity in tumor tissue. The Stemness Index Workflow (https://bioinformaticsfmrp.github.io/PanCanStem_Web/) applied the one-class algorithm to calculate tumor stem cells in the tumor sample. We first employed these two bioinformatic methods to evaluate tumor immune indices (immune score, stromal score, tumor purity, and ESTIMATE score) and stem cell index (mRNAsi score) and then standardized the value of indices ranging from 0 to 1.

### 2.5 Exploring the distinct clinical, molecular, and cellular features of subtypes

Based on clinical features such as age, stage, metastasis, Clark level, and so on, we first assessed the different distribution of clinical features among the cell death subtypes in TCGA_SKCM cohort. To be specific, melanoma patients may benefit from subtype-specific mutations as a treatment target. Therefore, we compared the different mutational frequencies, tumor mutation burden (TMB), and fraction genome altered (FGA) among subtypes of cancer. Moreover, to identify subtype-specific functional pathways, the differentially expressed genes in each subtype were annotated in terms of Gene Ontology (GO) biological processes (c5.bp.v7.1.symbols.gmt) by Gene Set Enrichment Analysis (GSEA). Furthermore, we referred to 10 tumor-associated gene sets, five immune-microenvironment gene signatures, and seven kinds of immune-checkpoint molecules which were collected and deposited in the “IOBR” package. Then, we used the single-sample gene set enrichment analysis (ssGSEA) method to estimate the enrichment scores among melanoma subtypes.

### 2.6 Chemotherapy response prediction

The Genomics of Drug Sensitivity in Cancer (GDSC; https://www.cancerrxgene.org/) database was used to explore the unique genomics of drug sensitivity across melanoma cell subtypes for identifying candidate agents that displayed variable effectiveness in cell death-related gene categorized clusters. In this database, over 1,000 genetically defined human cancer cell lines have been treated with a range of anti-cancer drugs (367 compounds). In total, 34 kinds of these cell lines are derived from cutaneous melanoma. We first used KNN machine learning to categorize these cell lines into corresponding cell death gene-classified subtypes and then examined the variations in the area under the curve (AUC) value of drug responses among these subtypes. After removing drugs with more than 20% missing values, only 219 compounds were selected for further analysis. To evaluate if there is a significant difference, Kruskal–Wallis test with a *p*-value of 0.05 was utilized.

### 2.7 Effect of drugs on A357 melanoma cell

#### 2.7.1 Chemicals

All chemicals were purchased from MedChemExpress (MCE, China) including ACY-1215 (no. HY-16026), tubastatin A (no. HY-13271A), and EHT 1864 (no. HY-16659).

#### 2.7.2 Cell lines and cell culture

The human melanoma cell line A375 was a gift from Associate Professor Naihong Yan from the Research Laboratory of Ophthalmology, West China Hospital, Sichuan University, China. All cells were cultured in Dulbecco’s modified Eagle’s medium (Gibco, USA), supplemented with 10% fetal bovine serum (Gibco, USA) and 1% penicillin/streptomycin mixture. The cells were maintained in a humidified incubator (37°C, 5% CO_2_, and 95% air).

#### 2.7.3 Cell viability assay

Cell viability was detected with cell counting kit-8 (CCK8, MCE, China). A375 cells were plated in 96-well plates at a density of 1 × 104 cells per well and treated with different concentrations of ACY-1215, tubastatin A, and EHT 1864 (total volume of 200 μl per well). After 24 and 48 h, 10 μl of CCK8 solution was added to each well of the plate and then incubated at 37°C in the dark for 1 h. The optical density was measured at 450 nm by a microplate reader (Bio-Rad, USA).

#### 2.7.4 Wound healing assay

The cells were seeded on six-well plates. After the cells have reached 100% confluence, the monolayer was scratched using a tip and washed with serum-free medium to remove detached cells. IC50 concentrations of ACY-1215, tubastatin A, and EHT 1864 were added to a serum-free medium for culture and photographed at 0, 24, and 48 h, respectively. The image-j software was applied to calculate the area of the wound.

#### 2.7.5 Cell apoptosis assay

Annexin V-FITC/propidium iodide (PI) Apoptosis kit (BD Biosciences, USA) was used to measure the apoptosis level of A375 cells treated with IC50 concentration of ACY-1215, tubastatin A, and EHT 1864, respectively. The cells were stained with Annexin V and PI according to the instructions and then analyzed by flow cytometry (Beckman, USA). Early apoptotic A375 cells showed annexin V-positive and PI-negative, and late apoptotic A375 cells were stained with both annexin V- and PI-positive. The results were presented as the percentage of apoptotic cells.

### 2.8 Pseudotime analysis of subtypes

The pseudotime analysis based on dimensionality reduction of graph learning was carried out by applying the reduced dimension function of the “Monocle” package. The dimension reduction approach was discriminative dimensionality reduction with trees, and we set the maximum number of components at five. Finally, the melanoma patients were landscaped and plotted using the plot cell trajectory function, with the color correlating to the cell death subtypes mentioned above.

### 2.9 Statistical analysis

Statistical analysis was entirely carried out by using the R package (v.4.0.3) and related packages. Unsupervised clustering was conducted by “MOVICS” package (21). The “survival” and “survivalROC” packages were used for survival analysis. ESTIMATE and molecular signal pathways were estimated and visualized by “IOBR” package (22). Pseudotime analysis was visualized by the “Monocle” package. The Spearman test was used to assess the correlation coefficient. For comparisons of two or more groups, the Wilcoxon and Kruskal–Wallis tests were used. Fisher’s exact or chi-square test was used to examine the relationship between subgroup and clinicopathological features.

## 3 Results

### 3.1 Melanoma collection and clustering

**Figure 1:**
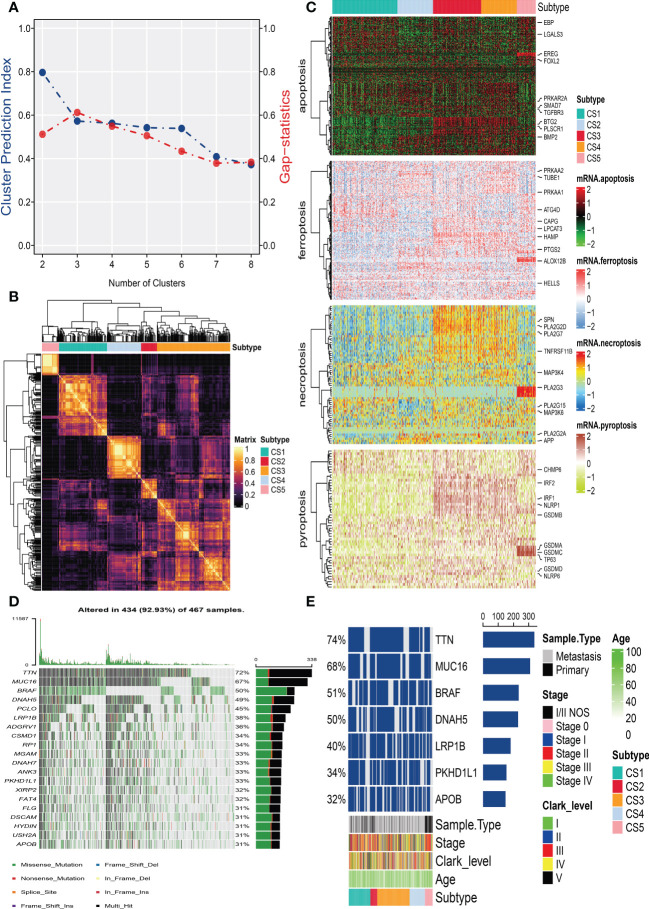
Identification of melanoma subtypes based on cell death-associated gene expression. **(A)** Calculating clustering prediction index (blue line) and Gaps-statistics (red line) in the cutaneous melanoma of The Cancer Genome Atlas data (TCGA_SKCM) cohort to determine the best cluster number. **(B)** Consensus heat map according to the findings of 10 integrative clustering methods with a cluster number of five. **(C)** Comprehensive heat map of distinctive molecular patterns across apoptosis, ferroptosis, necroptosis, and pyroptosis with annotation of genes. **(D)** Top 20 mutant genes in the TCGA_SKCM cohort. **(E)** Mutational OncoPrint of five identified melanoma subtypes in the TCGA_SKCM cohort.

After removing duplicated samples or samples without survival information, 462 melanoma patients were included in the TCGA_SKCM cohort. The meta-GEO cohort consisted of 412 melanoma patients, and the meta-immune response cohort consisted of 146 melanoma patients who received immune-checkpoint blockade therapy. A total of 570 genes that referred to four kinds of cell deaths (“apoptosis”, “ferroptosis”, “necroptosis”, and “pyroptosis”) were evaluated to explore the important clusters in the TCGA_SKCM cohort. In order to prove that these genes are consistent with cell death, we conducted GO enrichment analysis and found that these genes are positively activated in cell death-associated signal pathways such as regulation of the cysteine-type endopeptidase activity involved in the apoptotic process, regulation of apoptotic signaling pathway, and execution phase of apoptosis (**Supplementary Figure S1**). Next, CPI and gaps statistics uncovered that the optimum number of clusters *k* for clustering was three to five, and combined with the popular Clark level classifier for cutaneous melanoma, it has five classifications (**Figure 1A**). It should be highlighted that both CPI and gap statistics do not fall too much at *k* = 5. As a result of these considerations, *k* of 5 is selected as the ideal clustering number for further analysis. A consensus ensemble derived from 10 different clustering methods identified five robust subtypes of melanoma (**Figure 1B**). We defined these five clusters from CS1 to CS5. The complex cross-talk of four kinds of cell death expression may have a particular biological relevance that contributes to the heterogeneity of melanomas. Therefore, we generated a comprehensive heat map to present distinctive molecular patterns across apoptosis, ferroptosis, necroptosis, and pyroptosis (**Figure 1C**).

### 3.2 Somatic mutation in subtypes

**Figure 2:**
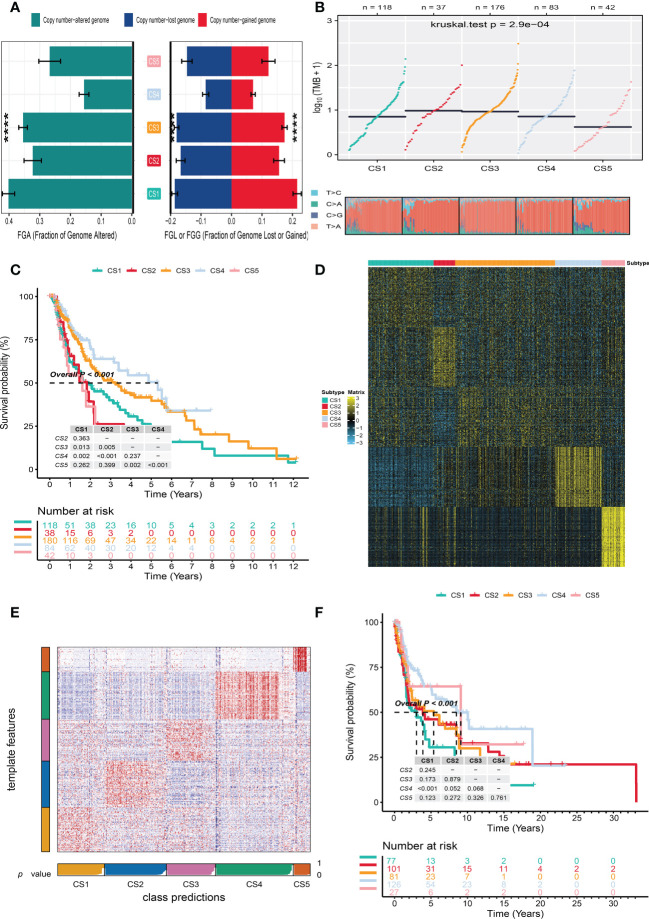
Independent verification of melanoma subtypes. **(A)** Bar plot of fraction genome altered among five identified melanoma subtypes in the cutaneous melanoma of The Cancer Genome Atlas data (TCGA_SKCM cohort). **(B)** Comparison of tumor mutation burden and single-nucleotide variants among five melanoma subtypes in the TCGA_SKCM cohort. **(C)** Kaplan–Meier survival curve of five melanoma subtypes in the TCGA_SKCM cohort. **(D)** Heat map of subtype-specific upregulated biomarkers for five melanoma subtypes in the TCGA_SKCM cohort. **(E)** Heat map of nearest template prediction in the meta-Gene Expression Omnibus (GEO) cohort using subtype-specific upregulated biomarkers identified from the TCGA_SKCM cohort. **(F)** Kaplan–Meier survival curve of five melanoma subtypes in the meta-GEO cohort. **** means p < 0.0001.

To identify the subtype-specific mutations in melanoma, the “maftools” package (23) was first employed to produce oncoPrint plots which illustrated the top 20 popular mutant genes including TTN (72%), MUC16 (67%), BRAF (50%), DNAH5 (49%), PCLO (45%) … (**Figure 1D**). The independent testing (Fisher’s exact or chi-square test) determined that seven genes (TTN, MUC16, BRAF, DNAH5, LRP1B, PKHD1L1, and APOB) have different mutant frequencies among these subtypes (**Figure 1E**). Among the frequently mutated genes, CS2 significantly harbored more mutations of TTN (86.5%; adj-*P* = 0.039), DNAH5 (54.1%; adj-*P* = 0.039), and APOB (37.8%; adj-*P* = 0.039) than the other subtypes, while CS3 and CS4 were significantly enriched in the mutations of BRAF (CS3: 57.4%; CS4: 60.2%; adj-*P* = 0.039), MUC16 (CS3: 76.1%; CS4: 71.1%; adj-*P* = 0.039), and LRP1B (48.3%; adj-*P* = 0.039) and were frequently mutated in CS3, whereas PKHD1L1 was significantly more mutated in CS2 than in other subtypes (40.5%; adj-*P* = 0.039) (**Table 1**). The FGA scores were then used to assess chromosomal instability, and we discovered that CS4 had superior chromosomal stability than the other subtypes, with a much lower fraction of genome lost or gained (**Figure 2A**). Moreover, TMB has arisen as an emerging biomarker for the prediction of numerous tumor types, prognosis, and immunotherapy responses. Therefore, TMB was contrasted among subtypes, and the Kruskal–Wallis test indicated that CS4 and CS3 have relatively higher TMB than the other subtypes (**Figure 2B**).

**Table 1:** Independent test between subtype and mutation.

Gene (mutated)	TMB	CS1	CS2	CS3	CS4	CS5	*p*-value	*p*-adjusted
FAT4	148 (32%)	40 (33.9%)	12 (32.4%)	58 (33.0%)	27 (32.5%)	11 (26.2%)	9.34e-01	9.34e-01
XIRP2	149 (33%)	37 (31.4%)	12 (32.4%)	64 (36.4%)	24 (28.9%)	12 (28.6%)	7.46e-01	7.87e-01
MUC16	309 (68%)	72 (61.0%)	23 (62.2%)	134 (76.1%)	59 (71.1%)	21 (50.0%)	4.39e-03	3.91e-02
HYDIN	143 (31%)	41 (34.7%)	11 (29.7%)	64 (36.4%)	19 (22.9%)	8 (19.0%)	7.52e-02	1.30e-01
PKHD1L1	154 (34%)	45 (38.1%)	15 (40.5%)	64 (36.4%)	25 (30.1%)	5 (11.9%)	1.16e-02	3.91e-02
APOB	148 (32%)	31 (26.3%)	14 (37.8%)	66 (37.5%)	31 (37.3%)	6 (14.3%)	1.44e-02	3.91e-02
USH2A	141 (31%)	33 (28.0%)	12 (32.4%)	58 (33.0%)	31 (37.3%)	7 (16.7%)	1.56e-01	2.12e-01
BRAF	234 (51%)	47 (39.8%)	14 (37.8%)	101 (57.4%)	50 (60.2%)	22 (52.4%)	6.66e-03	3.91e-02
DNAH7	158 (35%)	35 (29.7%)	13 (35.1%)	68 (38.6%)	31 (37.3%)	11 (26.2%)	3.94e-01	4.40e-01
DNAH5	230 (50%)	51 (43.2%)	20 (54.1%)	107 (60.8%)	38 (45.8%)	14 (33.3%)	3.13e-03	3.91e-02
RP1	155 (34%)	38 (32.2%)	13 (35.1%)	70 (39.8%)	28 (33.7%)	6 (14.3%)	2.99e-02	6.31e-02
ANK3	154 (34%)	35 (29.7%)	15 (40.5%)	62 (35.2%)	34 (41.0%)	8 (19.0%)	9.23e-02	1.35e-01
DSCAM	141 (31%)	37 (31.4%)	14 (37.8%)	61 (34.7%)	23 (27.7%)	6 (14.3%)	8.25e-02	1.31e-01
PCLO	209 (46%)	48 (40.7%)	19 (51.4%)	94 (53.4%)	36 (43.4%)	12 (28.6%)	2.55e-02	6.06e-02
FLG	149 (33%)	40 (33.9%)	7 (18.9%)	62 (35.2%)	29 (34.9%)	11 (26.2%)	3.10e-01	3.68e-01
CSMD1	165 (36%)	46 (39.0%)	12 (32.4%)	74 (42.0%)	24 (28.9%)	9 (21.4%)	5.77e-02	1.10e-01
LRP1B	181 (40%)	46 (39.0%)	13 (35.1%)	85 (48.3%)	28 (33.7%)	9 (21.4%)	1.13e-02	3.91e-02
TTN	337 (74%)	81 (68.6%)	32 (86.5%)	140 (79.5%)	59 (71.1%)	25 (59.5%)	1.39e-02	3.91e-02
MGAM	156 (34%)	36 (30.5%)	12 (32.4%)	71 (40.3%)	27 (32.5%)	10 (23.8%)	2.23e-01	2.82e-01

### 3.3 Survival analysis and validation of meta-GEO cohort

In the TCGA_SKCM cohort, 118 melanoma patients were classified into the CS1 subgroup, CS2 had 39 melanoma patients, and the CS3, CS4, and CS5 subgroups respectively consisted of 180, 84, and 42 patients. Among these subgroups, CS4 has the best survival prognosis than the other subgroups (overall survival, *P* < 0.001) (**Figure 2C**). The stratified survival analysis revealed that CS4 has a longer survival time than the patients in the CS1 (*P* = 0.002), CS2 (*P* < 0.001), and CS5 (*P* < 0.001) subtypes. Furthermore, we also discovered that CS3 has a better prognosis than the patients in CS1 (*P* = 0.013), CS2 (*P* = 0.005), and CS5 (*P* = 0.002), whereas there was no statistical significance compared with the CS4 subgroup. Next, the independent testing for a different distribution of clinical features indicated that these classifications were substantially correlated to overall survival time, age, AJCC stage, Clark level, tumor stage, and metastatic sample type (**Table 2**).

**Table 2:** Summary of clinical features.

*n*	level	CS1	CS2	CS3	CS4	CS5	*p*	Test
		118	39	180	84	42		
Futime (median, IQR)		322.26 (167.61, 892.35)	303.12 (164.40, 590.76]	583.44 (263.94, 1,114.92]	694.38 (334.53, 1,394.07]	183.90 (131.31, 330.90]	<0.001	Nonnorm
Fustat (%)	0	51 (43.2)	19 (48.7)	94 (52.2)	51 (60.7)	26 (61.9)	0.085	Exact
	1	67 (56.8)	20 (51.3)	86 (47.8)	33 (39.3)	16 (38.1)		
Age (median, IQR)		61.00 (48.25, 72.00]	63.00 (54.00, 71.50]	55.50 (45.00, 66.00]	56.50 (46.00, 71.50]	60.00 (56.00, 75.75)	0.014	Nonnorm
Metastasis_Stage (%)	M0	101 (95.3)	36 (97.3)	165 (93.2)	71 (94.7)	40 (95.2)	0.933	Exact
	M1	5 (4.7)	1 (2.7)	12 (6.8)	4 (5.3)	2 (4.8)		
LymphNode_Stage (%)	N0	62 (55.9)	21 (55.3)	88 (49.7)	36 (47.4)	24 (57.1)	0.389	Exact
	N1	17 (15.3)	4 (10.5)	31 (17.5)	15 (19.7)	6 (14.3)		
	N2	7 (6.3)	4 (10.5)	25 (14.1)	9 (11.8)	4 (9.5)		
	N3	16 (14.4)	6 (15.8)	21 (11.9)	12 (15.8)	1 (2.4)		
	NX	9 (8.1)	3 (7.9)	12 (6.8)	4 (5.3)	7 (16.7)		
Stage (%)	I/II NOS	2 (1.8)	1 (2.7)	3 (1.8)	3 (4.0)	1 (2.5)	<0.001	Exact
	Stage 0	3 (2.7)	2 (5.4)	0 (0.0)	1 (1.3)	0 (0.0)		
	Stage I	15 (13.6)	2 (5.4)	39 (23.6)	20 (26.7)	2 (5.0)		
	Stage II	47 (42.7)	17 (45.9)	42 (25.5)	10 (13.3)	24 (60.0)		
	Stage III	38 (34.5)	14 (37.8)	70 (42.4)	37 (49.3)	11 (27.5)		
	Stage IV	5 (4.5)	1 (2.7)	11 (6.7)	4 (5.3)	2 (5.0)		
Tumor_Stage (%)	T1	8 (7.2)	5 (13.5)	28 (16.3)	23 (30.7)	1 (2.4)	<0.001	Exact
	T2	19 (17.1)	1 (2.7)	39 (22.7)	15 (20.0)	3 (7.3)		
	T3	23 (20.7)	8 (21.6)	37 (21.5)	16 (21.3)	6 (14.6)		
	T4	48 (43.2)	19 (51.4)	43 (25.0)	13 (17.3)	30 (73.2)		
	Tis	3 (2.7)	3 (8.1)	0 (0.0)	1 (1.3)	0 (0.0)		
	TX	10 (9.0)	1 (2.7)	25 (14.5)	7 (9.3)	1 (2.4)		
Clark_level (%)	I	2 (2.2)	3 (10.7)	0 (0.0)	0 (0.0)	0 (0.0)	0.002	Exact
	II	3 (3.4)	0 (0.0)	10 (8.4)	3 (5.7)	2 (6.5)		
	III	16 (18.0)	1 (3.6)	36 (30.3)	19 (35.8)	5 (16.1)		
	IV	49 (55.1)	17 (60.7)	58 (48.7)	25 (47.2)	18 (58.1)		
	V	19 (21.3)	7 (25.0)	15 (12.6)	6 (11.3)	6 (19.4)		
Race (%)	Asian	3 (2.6)	0 (0.0)	6 (3.4)	0 (0.0)	3 (7.5)	0.11	Exact
	Black or African American	0 (0.0)	0 (0.0)	0 (0.0)	1 (1.2)	0 (0.0)		
	White	113 (97.4)	38 (100.0)	170 (96.6)	82 (98.8)	37 (92.5)		
Sample.Type (%)	Metastasis	86 (72.9)	24 (61.5)	161 (89.4)	81 (96.4)	7 (16.7)	<0.001	Exact
	Primary	32 (27.1)	15 (38.5)	19 (10.6)	3 (3.6)	35 (83.3)		
Sex (%)	Female	42 (35.6)	18 (46.2)	57 (31.7)	40 (47.6)	18 (42.9)	0.093	Exact
	Male	76 (64.4)	21 (53.8)	123 (68.3)	44 (52.4)	24 (57.1)		
Neoplasm_Status (%)	Tumor-free	47 (43.5)	18 (48.6)	77 (45.3)	48 (58.5)	30 (76.9)	0.003	Exact
	With tumor	61 (56.5)	19 (51.4)	93 (54.7)	34 (41.5)	9 (23.1)		

**Figure 3:**
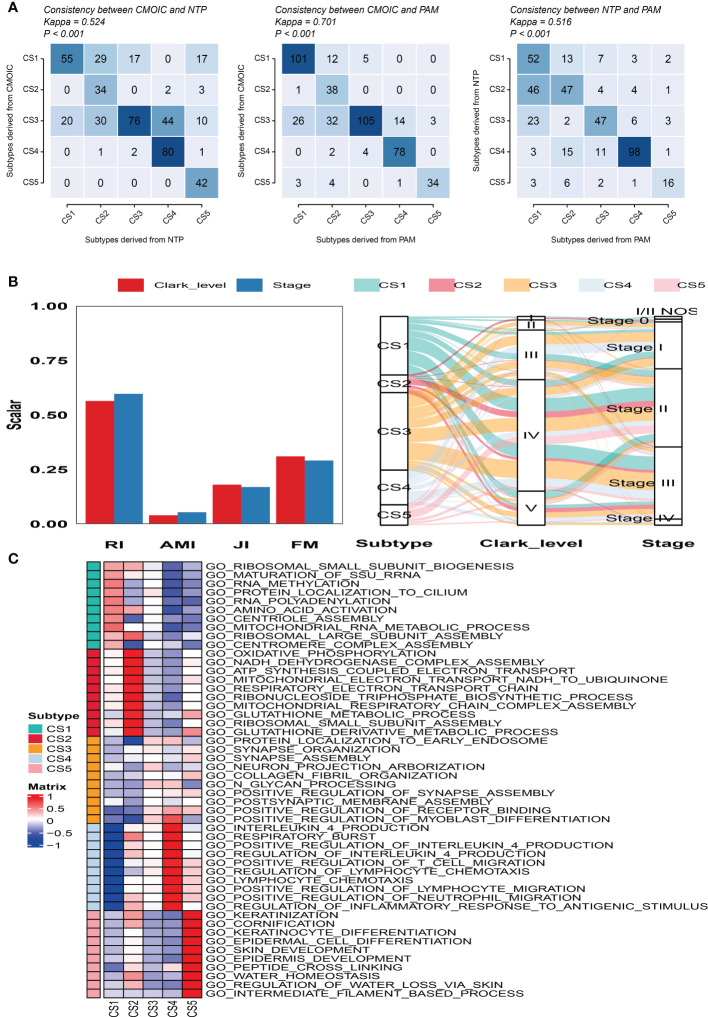
Consistency evaluation of different prediction approaches. **(A)** Consistency heat map using Kappa statistics among different prediction approaches. **(B)** Agreement of the predicted five subtypes of melanoma with Clark level and tumor stage classification in the cutaneous melanoma of The Cancer Genome Atlas data cohort. **(C)** Heat map of subtype-specific functional pathways based on upregulated genes for five identified melanoma subtypes.

To validate the robustness of the cell death clustering, we first applied the “limma” approach to identify the biomarkers for each subtype which are picked from a list of the most up-expressed genes ordered by log_2_ fold change (100 biomarkers for each subtype). These biomarkers must satisfy the significance level (adjusted *P*-value 0.05 and log_2_ fold change > 0) and not overlap with any other subtype-identified biomarkers. The heat map visualized the expression of 100 biomarkers for identifying subtypes of melanoma in the TCGA_SKCM cohort (**Figure 2D** and **Supplementary Table S2**). Subsequently, based on the template heat map (**Figure 2E**), nearest template prediction (NTP) was conducted to predict a sample category in the meta-GEO cohort. The Kaplan–Meier survival curve of the predicted five subgroups in the meta-GEO cohort suggested that CS4 was also the best subgroup for prognosis than the others (overall survival, *P* < 0.001) (**Figure 2F**). To explore the potential prognostic mechanism in the CS4 subgroup, we mapped the 100 biomarkers in the CS4 subgroup to the STRING website and screened the corresponding proteins. According to the protein–protein interaction (PPI) network analysis (**Supplementary Figure S2A**), seven hub genes including CD79A, CD79B, CD19, CCR7, CD40LG, SELL, and ZAP70 (degree ≥20) were identified in the CS4 subtype. These hub genes contribute to the activation of primary B-lymphocytes as well as the proliferation of T-cell and cytokine production. The Wilcoxon tests indicated that most of the hub genes (six of seven) were differentially expressed in the TCGA–Genotype Tissue Expression dataset (**Supplementary Figure S2B**) (24). Furthermore, to prove the results from the database, we also used immunohistochemistry to confirm that these hub genes were also differentially increased in melanoma compared with normal skin samples (**Supplementary Figure S2C**). Lastly, to test the comparability and repeatability of acquired subtypes across discovery and validation cohorts, the in-group proportion (IGP) statistic will be utilized (25). The values of the IGP scale are from 0 to 1. Most of the IGPs for each subtype in the discovery and validation cohorts were higher than 0.7 (**Supplementary Table S3**), which indicates a repeatable patient partition for that subtype. Furthermore, several different predictive approaches like partition around medoids classifier, NTP, and consensus ensemble algorithms were performed for accurate classification (21, 26, 27). The Kappa statistics manifested that there is a relatively high consistency among these methods (**Figure 3A**). Presently, melanomas have traditional classifications such as AJCC stage and Clark level. It is critical to verify the consistency of emerging subtypes with traditional classifications in order to represent the robustness of clustering analysis. Therefore, adjusted mutual information (AMI), Fowlkes–Mallows (FM), Jaccard Index (JI), and Rand Index (RI) were applied to evaluate the similarity between the current subtypes and other well-established subtypes (21). All of these statistics were scaled ranging from 0 to 1, and the higher the number is, the closer the two appraisals are. We observed a moderated agreement in RI, JI, and FM estimation and a slight similarity in AMI calculation. The alluvial diagram also shows the agreement between the AJCC stage, Clark level, and the present subtypes (**Figure 3B**).

### 3.4 Microenvironment, molecular features of subtypes

Firstly, GSEA was conducted for each subtype to identify subtype-specific functional pathways according to their corresponding upregulated genes. Based on the significance threshold (*P*-value < 0.05 and adjusted *P*-value < 0.25) and without any overlapped pathways identified for other subtypes, we observed that CS1 mainly enriched in protein synthesis and post-translational modifications such as ribosomal subunit biogenesis, assembly, and methylation. CS2 was significantly involved in energy metabolism and mitochondrial function like oxidative phosphorylation, NADH dehydrogenase complex assembly, and ATP synthesis coupled electron transport. The subtypes of CS3 and CS4 mainly referred to immune response and inflammatory regulation such as positive regulation of receptor binding, interleukin 4 production, and positive regulation of T cell migration. The CS5 subtype was significantly enriched in pathways associated with skin development, keratinization, and cornification (**Figure 3C**).

**Figure 4:**
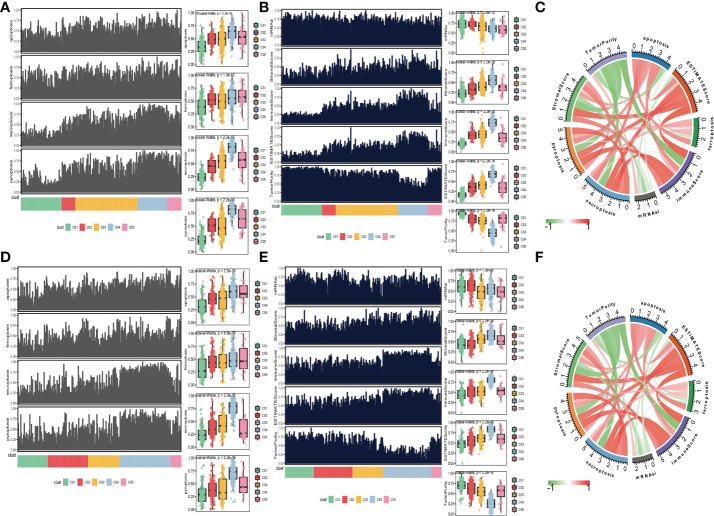
Association between cell deaths and tumor microenvironment. **(A)** Distribution of four cell death indices (apoptosis, ferroptosis, necroptosis, and pyroptosis) among five identified melanoma subtypes in the cutaneous melanoma of The Cancer Genome Atlas data (TCGA_SKCM) cohort. **(B)** Distribution of tumor microenvironment-related predictors (immune score, stromal score, tumor purity, ESTIMATE score, and mRNAsi score) among five identified melanoma subtypes in the TCGA_SKCM cohort. **(C)** Correlation coefficients between cell death indices and tumor microenvironment-related predictors in the TCGA_SKCM cohort. **(D)** Distribution of four cell death indices among five identified melanoma subtypes in the meta-Gene Expression Omnibus (GEO) cohort. **(E)** Distribution of tumor microenvironment-related predictors among five identified melanoma subtypes in the meta-GEO cohort. **(F)** Correlation coefficients between cell death indices and tumor microenvironment-related predictors in the meta-GEO cohort.

Moreover, to explore the relationship between cell deaths and tumor microenvironment, cell death indices (apoptosis, ferroptosis, necroptosis, and pyroptosis) and tumor microenvironment-related predictors (immune score, stromal score, tumor purity, ESTIMATE score, and mRNAsi score) were calculated and plotted. The distribution of cell death indices for each subtype in the TCGA_SKCM cohort is illustrated in **Figure 4A**. Kruskal–Wallis test determined that melanoma patients in the CS4 subtype had the highest cell death score than the other subtypes, whereas the cell death scores of CS1 and CS2 were located at a much lower level. Similarly, we surprisingly observed that the CS4 subtype had a superior immune score, stromal score, and ESTIMATE score than the other subtypes (**Figure 4B**), while tumor purity and mRNAsi score in CS4 were the minima among subtypes. The correlation analysis discovered that four kinds of cell death (apoptosis, ferroptosis, necroptosis, and pyroptosis) were not only positively associated with each other but also have a close positive correlation with immune score, stromal score, and ESTIMATE score. There were negative associations between cell deaths with tumor purity and mRNAsi score (**Figure 4C**). To prove our observation, similar analyses were performed in the meta-GEO cohort. Similar distribution trends and statistical results were observed in cell death indices (**Figure 4D**) and tumor microenvironment-related predictors (**Figure 4E**). The circle plot manifested similar correlation coefficients compared with the TCGA_SKCM cohort (**Figure 4F**).

**Figure 5:**
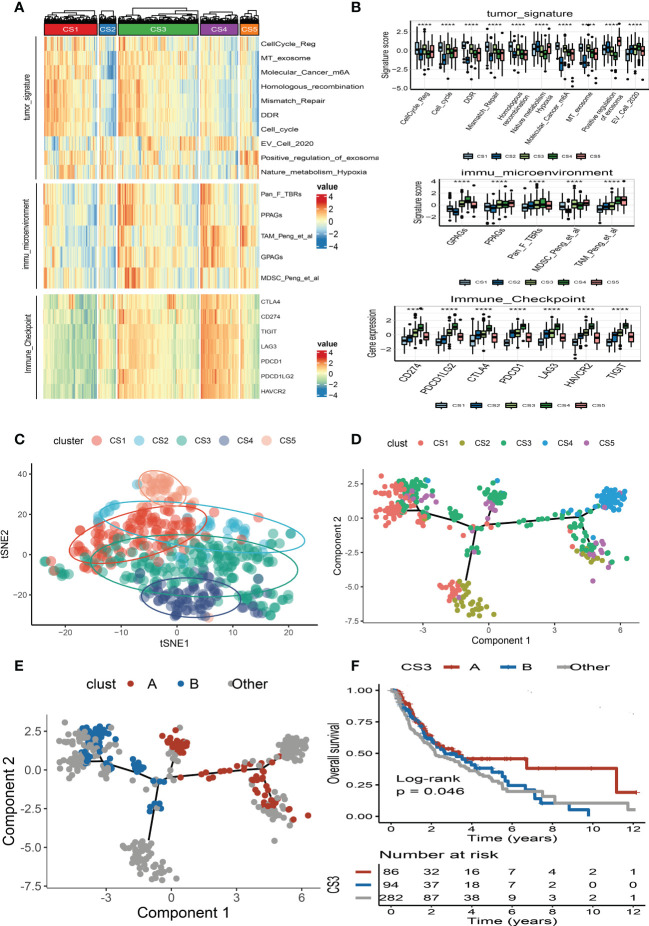
Landscape of cell deaths for five identified melanoma subtypes. **(A)** Comprehensive heat map of tumor-associated pathways, immune-microenvironment signatures, and expression of immune-checkpoint molecules among five identified melanoma subtypes. **(B)** Box plot of tumor-associated pathways, immune-microenvironment signatures, and expression of immune-checkpoint molecules among subtypes. **(C)** t-SNE plot for melanoma subtype distribution. **(D)** Graph learning-based dimensionality reduction plot of melanoma subtypes; each color represents a subtype corresponding to the previously defined subtype. **(E)** The intra-cluster heterogeneity within CS3 subtype, which was further divided into two subgroups according to their location in graph learning. **(F)** Kaplan–Meier survival curve of subgroups A and B in CS3 subtype. **** means p < 0.0001.

We also produced a heat map to comprehensively depict the distinct features of tumor-associated pathways, immune-microenvironment signatures, and expression of immune-checkpoint molecules (**Figure 5A**). The Kruskal–Wallis test detected that tumor-associated pathways such as cell cycle, m6A regulation, DNA damage response (DDR), mismatch repair, and metabolism hypoxia were positively enriched in the CS1 subtype (**Figure 5B**). As for the previous report, the immune microenvironment signatures and immune-checkpoint molecules are where the subtypes of CS3 and CS4 had the highest expression values compared to the others (**Figure 5B**).

### 3.5 Pseudotime analysis of subtypes

To better visualize the five clusters based on unsupervised clustering and identify the underlying mechanisms of individual patient distributions, we performed the t-SNE algorithms to investigate and illustrate cluster categorization across samples. The two-dimensional tSNE plot showed that melanoma patients in the TCGA_SKCM cohort can be distinctively partitioned into five clusters (**Figure 5C**). In addition, the cell death-associated gene expression was subjected to the graph learning-based dimensionality reduction approach. Individual patients were placed on a manifold with sparse tree structures, and the landscape of melanoma for cell deaths was established. Most patients were classified into separate clusters at the end of the branch, such as CS1, CS2, CS4, and CS5 subtypes, which were consistent with our previously described unsupervised clustering (**Figure 5D**). Individual patient placed in the landscape indicates the general features of cell death in the relevant subtype. In the cell death landscape, for example, the death-active subtype CS4 and death-inactive subtypes CS1 and CS2 were dispersed at the opposite end of the horizontal axis (**Figure 5D**). As a result, we speculated that the cell death landscape’s vertical axis indicates a different status of cell death for patients.

**Figure 6:**
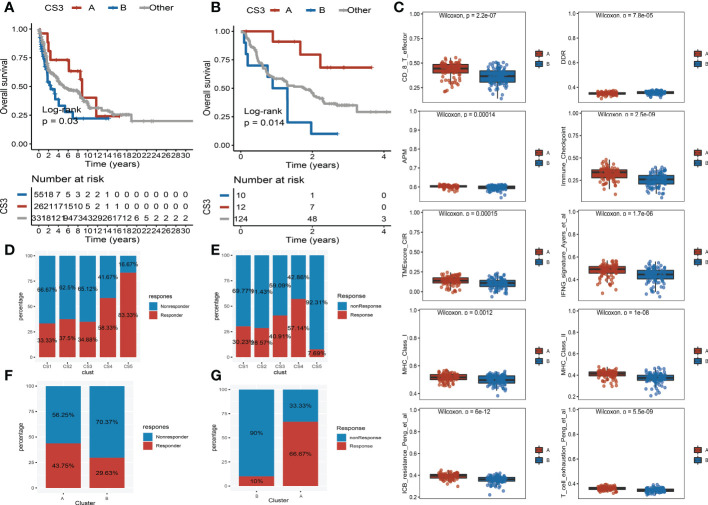
Validation of heterogeneity within CS3 subtype and immunotherapy response. **(A)** Kaplan–Meier survival curve of subgroups A and B in CS3 subtype at the meta-Gene Expression Omnibus (GEO) cohort. **(B)** Kaplan–Meier survival curve of subgroups A and B in CS3 subtype at the meta-immune response cohort. **(C)** Box plot of CD8 T cell effector, DNA damage response, antigen processing and presenting machinery, immune-checkpoint, tumor microenvironment score, INFG signature, MHC classes I and II, ICB resistance, and T cell exhaustion between subgroups **(A)** and **(B, D)** Immunotherapy response rate of five identified melanoma subtypes in the cutaneous melanoma of The Cancer Genome Atlas data (TCGA_SKCM) cohort. **(E)** Immunotherapy response rate of five identified melanoma subtypes in the meta-immune response cohort. **(F)** Rate of immunotherapy response for subgroups A and B in CS3 subtype at the TCGA_SKCM cohort. **(G)** Rate of immunotherapy response for subgroups A and B in CS3 subtype at the meta-immune response cohort.

Moreover, we observed that the samples of the CS3 subtype have scattered distributions on both sides of the vertical axis in the landscape. Therefore, we believed that a significant intra-cluster heterogeneity existed in the CS3 subtype. Based on the vertical axis in the landscape, we successfully divided the CS3 cluster into two subgroups (group A and group B) (**Figure 5E**). The Kaplan–Meier (KM) survival plots manifested that group A in the CS3 subtype had good survival than group B with log-rank *P* = 0.046 (**Figure 5F**). To assess the classification robustness, the CS3 subtype in the meta-GEO cohort and meta-immune response cohort was correspondingly classified into two subgroups. The KM survival curves revealed similar outcomes that group A in the CS3 subtype had a longer survival time than group B regardless of the meta-GEO cohort (**Figure 6A**) and meta-immune response cohort (**Figure 6B**). Interestingly, the two subgroups of the CS3 cluster identified by the cell death landscape not only have distinct survival outcomes but also were correlated with different molecular features—for example, patients in group A had a positive enrichment in CD8 T cell effector, antigen processing and presenting machinery (APM), immune-checkpoint, TME score, INFG signature, MHC classes I and II, ICB resistance, and T cell exhaustion (**Figure 6C**), whereas group B was much more inclined to cancer hallmark pathways like DDR (**Figure 6C**).

### 3.6 Prediction of immunotherapy and chemotherapy response

Because of the distinct distribution of expression of immune-checkpoint molecules among subtypes, we speculated that our classifications based on cell deaths can be applied to predict immunotherapy response in patients of melanoma. To prove our guesses, the melanoma patients who received immunotherapy in the TCGA_SKCM cohort and the meta-immune response cohort were classified into five categories accordingly. We surprisingly uncovered that the response rate of the CS4 subgroup was relatively higher than the other subgroups no matter if in the TCGA_SKCM cohort or the meta-immune response cohort. Patients in the CS4 subgroup have a 58.33% immunotherapy response in the TCGA_SKCM cohort (**Figure 6D**). Meanwhile, in the meta-immune response cohort, the CS4 subgroup has the highest response rate (57.14%) than the other subgroups (**Figure 6E**) Moreover, the CS3 subtype had more diversified cell death and immune characteristics with moderate immunotherapy response. A subdivided analysis indicated that the enrichment score of immune-checkpoints in group A was higher than in group B in the CS3 subtype. Thus, we also explored the immune response rate between group A and group B. We observed that group A had a larger response rate than group B regardless of the TCGA_SKCM cohort and the meta-immune response cohort. In the TCGA_SKCM cohort, patients in group A have a 43.75% response rate *versus* 29.63% in group B (**Figure 6F**). At the same time, group A in the meta-immune response cohort has a larger proportion response rate than group B (66.67% *vs*. 10%) (**Figure 6G**).

**Figure 7:**
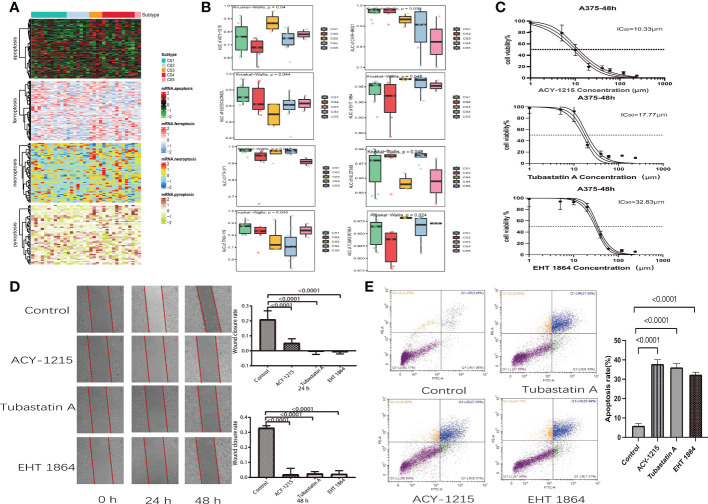
Drug identification in cell death-associated gene classified clusters of melanoma. Immunotherapeutic response and potential compounds. **(A)** Heat map of the differential expression of cell death-associated gene classified clusters in melanoma cells at the Genomics of Drug Sensitivity in Cancer database. **(B)** Box plot of the area under the curve of ACY-1215, CHIR-99021, EHT-1864, ELESCLOMOL, FTI-277, NILOTINIB, TUBASTATIN A, and TWS-119 among five clusters. **(C)** Cell viability curves and estimated IC50 values of ACY-1215, tubastatin A, and EHT-1864. **(D)** Wound healing assay in A375 cells was performed after treatment with ACY-1215 (10.33 μm), tubastatin A (17.77 μm), and EHT-1864 (32.83 μm) at a 48-h recovery period. **(E)** Flow cytometry analysis of A357 cells which were stained with Annexin V-FITC and propidium iodide after 48 h of ACY-1215 (10.33 μm), tubastatin A (17.77 μm), and EHT-1864 (32.83 μm) treatment.

Scientists are struggling to discover new potential compounds for melanoma due to resistance to standard chemotherapeutics (28). To investigate candidate agents that displayed variable effectiveness in cell death gene categorized clusters, the GDSC database was used to test the prediction model (**Figure 7A**). The AUC values of drug responses were compared within clusters. The GDSC database has 367 compounds in total. Only 219 compounds were evaluated on 34 kinds of melanoma cell lines that were utilized in the analysis. The Kruskal–Wallis tests detected eight compounds, including ACY-1215, CHIR-99021, EHT-1864, ELESCLOMOL, FTI-277, nilotinib, tubastatin A, and TWS-119, that were distinctly affected in cell death gene categorized clusters (**Figure 7B**). Furthermore, we observed that the AUC values of ACY-1215, EHT-1864, and tubastatin A were significantly lower in cluster 2. Elesclomol and nilotinib were significantly lower in cluster 3. In cluster 5, the AUC values of CHIR-99021 and FTI-277 were much lower than in the other clusters. Furthermore, TWS-119 was much lower in cluster 3. The lower AUC of the drug responses means being more sensitive to drug therapy.

### 3.7 Effect of ACY-1215, EHT-1864, and tubastatin A on A357 melanoma cell

We discovered that melanoma patients with death-inactive subtypes are more sensitive to ACY-1215, EHT-1864, and tubastatin A. Therefore, these drugs were selected for cell experiments. Firstly, the CCK8 test was used to measure the cell viability and IC50 values of drugs on A357 melanoma cells. We found that both ACY-1215 and tubastatin A can effectively kill A357 cells with lower concentrations. The IC50 values of ACY-1215, tubastatin, and EHT-1864 were 10.33, 17.7, and 32.83 μm, respectively (**Figure 7C**). Furthermore, the wound healing assay indicated that ACY-1215, tubastatin, and EHT-1864 can significantly attenuate the cell migration of A357 after incubation for 24 and 48 h (**Figure 7D**). The effect of ACY-1215, tubastatin, and EHT-1864 on A357 cell apoptosis was studied using flow cytometry. The results show that these drugs can obviously increase A357 cell apoptosis (**Figure 7E**).

## 4 Discussion

The prevalence of advanced melanoma has risen steadily over the last few decades. However, treatment regimens have markedly increased in recent years as a result of an improved understanding of the underlying tumorigenesis and signal transduction pathways associated with its pathological process—for example, cancer immunotherapies focused on checkpoint inhibitors have reached significant clinical success. However, its usage is severely limited to the fact that just one-third of patients with most forms of melanoma respond to these inhibitors (29). Several research findings tried to integrate cell death induction with immune-checkpoint inhibitors, resulting in synergistically enhanced antitumor activity, even in checkpoint inhibitor-resistant tumors (17). Under this scenario, researchers proposed a novel notion, immunogenic cell death, which might be induced by radiation, tumor vaccination, immunotherapy, or some form of chemotherapy (30). Immunogenic cell death (ICD) was previously known as immunogenic apoptosis since most kinds of ICD are caused by apoptosis. As the understanding of cell death processes has increased, many nonapoptotic cell deaths have been characterized in recent years. Three well-studied nonapoptotic cell deaths include necroptosis, pyroptosis, and ferroptosis (31–33). As a consequence, in this study, we examined the ICD-associated RNA-seq data of melanoma patients for exploration, independent verification, and comprehensive classification of five reproducible melanoma subtypes in multiple cohort studies, which put a spotlight on cancer classification for frontline therapeutic approaches.

Various gene expression-based biomarkers in the literature can categorize individuals who are at risk and who can benefit from individualized treatments, but their correctness and predictive ability stay restricted (34–38). Nevertheless, the majority of this research relied on the differential study of genomic or transcriptome characteristics rather than biological processes (39). In the current research, we first collected the genes referred to “apoptosis”, “ferroptosis”, “necroptosis”, and “pyroptosis” processes. Moreover, to prove that these genes are consistent with cell death, we conducted GO enrichment analysis and found that these genes are positively activated in cell death-associated signal pathways. Next, we investigated the variations of specific melanoma subtypes based on cell death gene expression and its relationship to tumor genetic mutations as well as the immunological environment. We found that patients in the high-risk subgroups (CS1, CS2, and CS5) with low cell death indices were associated with high Clark levels and tumor stage. These groups also manifested worse overall survival. The substantial connection of cell death with clinical characteristics and survival outcomes suggests that cell death is effective in melanoma patients’ prognosis. Furthermore, we observed that patients in CS3 and CS4 subgroups with good prognoses were significantly enriched in mutations of BRAF and MUC16. Consistently, previous research has proven that BRAF mutation in melanoma patients indicates a higher overall and relative survival rate (40, 41). Several studies indicate that a BRAF mutant could be identified by host immunity and may play a role in anticancer immune responses (42). Erkes et al. recently reported that BRAF inhibitors promoted pyroptosis in anticancer immune responses, pointing to novel treatment approaches for refractory melanoma (43). Furthermore, the mutation of MUC16 was linked to better overall survival in both NSCLC and melanoma (44). Wang et al. have previously proven that MUC16 mutations are mainly enriched in immune-associated pathways and are favorably linked with T-cell activation, which may enhance the prognosis of melanoma patients (45).

Recent research has revealed strong links between cell death and anti-cancer immunity—for example, the tumor-infiltrated CD8+ T cells are thought to induce tumor cell death *via* perforin-granzyme and Fas-FasL. The new mechanism, however, has illustrated that CD8+ T cells suppress tumor growth by inducing ferroptosis, pyroptosis, and necroptosis, which prompted a reconsideration of the relationship between tumor cell death and immune activation (17). In this study, we observed that apoptosis, necroptosis, pyroptosis, and ferroptosis positively correlated with a highly infiltrated immune microenvironment and highly expressed immune-checkpoint genes, which was consistent with many previous tumor cell death research (46–48). Furthermore, compared with patients in the low-cell-death group, patients in the high-cell-death groups (CS3 and CS4) had a lower expression of tumor-associated molecular pathways and higher enrichment of immune response biological pathways. A variation in the functional immunological network, as previously reported, can cause disruptions in anti-cancer response, immunoediting, and cancer cell escape (49). This heterogeneity of cell death patterns combined with distinct tumor immune microenvironments offers a once-in-a-lifetime chance to develop targeted therapeutics (50). Patients with high infiltration of the immune microenvironment and highly expressed checkpoint target genes are more particularly sensitive to immune-checkpoint inhibitors. We discovered that melanoma patients in CS3 and CS4 subtypes with a high expression of checkpoint target genes are more sensitive to checkpoint inhibitor therapy. However, basic unsupervised clustering analysis for patient subtyping frequently failed to identify intra-cluster interactions and did not reveal the overall structure of the patient distribution (51)—for example, a subtype of CS3 had similar prognosis and clinical outcomes compared with the CS4 subtype. However, the CS3 subtype manifested a more sophisticated heterogeneity of the immune microenvironment and had a lower chromosomal instability than the CS4 subtype. To address these issues, we used graph learning algorithms to identify the tree topologies of cell death profiles in patients, which supplied additional information to clustering analysis and provided fresh insight into the complicated melanoma cell death landscape. We found that the CS3 subtype can successfully cluster into two subgroups (group A and group B) with different survival outcomes, molecular features, and distinct responses of immune-checkpoint inhibitor therapies.

The biological differences between the five categories may signal the necessity for distinct treatment strategies. Based on the GDSC database, we identified eight compounds that were distinctly affected in cell death gene categorized clusters. ACY-1215 and tubastatin A are selective inhibitors of HDAC6, which are required for the proliferation and metastasis of melanoma cells (52, 53). Previous studies have revealed that HDAC6 inhibitors might suppress the growth of a panel of human melanoma cell lines and could be a potential strategy for melanoma therapy, even resolving vemurafenib resistance (53, 54). In our research, we observed that ACY-1215, tubastatin A, and EHT 1864 are more sensitive to the death-inactive subtypes (CS1, CS2, and CS5) of melanoma patients. Cell experiments also confirmed that ACY-1215, tubastatin A, and EHT 1864 can effectively kill A357 cells *via* apoptosis. Several previous experiments have proven that selective inhibitors of HDAC6 synergistically improve anticancer activity *via* induction of tumor cell death (55, 56). Elesclomol is an investigational agent that causes oxidative stress, mitochondrial-induced apoptosis in tumor cells, and synergies with taxanes in tumor models (57). In phase II clinical trial of patients with metastatic melanoma, elesclomol with paclitaxel was observed to improve progression-free survival compared with paclitaxel alone (58). The phase II Tasigna Efficacy in Advanced Melanoma trial suggested that nilotinib, a KIT-selective tyrosine kinase inhibitor, may be an effective therapy option for individuals with KIT-mutated advanced melanoma (59). Moreover, CHIR 99021 is a GSK3 kinase inhibitor that can significantly enhance TNF and IFN production, natural cytotoxicity, and antibody-dependent cellular cytotoxicity for effective cancer immunotherapy (60). In conclusion, it is reasonable to speculate that these chemicals may induce various kinds of cell death and can be employed as precise therapies for some death-inactive melanoma subtypes.

## 5 Conclusion

In summary, we systematically landscaped five cell death subtypes in melanoma that were connected with distinct anti-cancer immunity and found vastly differing patterns in tumor genetic mutation, immune-checkpoint inhibitors, tumor-infiltrating environment, functional orientation, and, most importantly, clinical outcomes. This research gives a conceptual framework for understanding melanoma cell death and immune response and has practical implications for individualized immunotherapy and chemotherapy.

## Data availability statement

The datasets presented in this study can be found in online repositories. The names of the repository/repositories and accession number(s) can be found in the article/**Supplementary Material**.

## Author contributions

QW was in charge of writing. RW and XW were responsible for the duty of editing. Y-pD was responsible for project administration. All authors contributed to the article and approved the submitted version.

## Conflict of interest

The authors declare that the research was conducted in the absence of any commercial or financial relationships that could be construed as a potential conflict of interest.

## Publisher’s note

All claims expressed in this article are solely those of the authors and do not necessarily represent those of their affiliated organizations, or those of the publisher, the editors and the reviewers. Any product that may be evaluated in this article, or claim that may be made by its manufacturer, is not guaranteed or endorsed by the publisher.

## Supplementary material

Supplementary Figure 1 The Gene Ontology enrichment of cell death- related genes.

Supplementary Figure 2 Protein–protein interaction (PPI) network of CS4 subtype biomarkers. **(A)** The PPI network of biomarkers and seven hub genes. **(B)** Box plot of hub genes between tumor and normal skin groups in the Cancer Genome Atlas–Genotype Tissue Expression dataset. **(C)** Immunohistochemical images of hub genes in melanoma tissues and normal samples.

Supplementary Table 1 List of gene sets.

Supplementary Table 2 Gene biomarkers for identifying the subtypes of melanoma in the cutaneous melanoma of The Cancer Genome Atlas data cohort.

Supplementary Table 3 The in-group proportion statistic in the cutaneous melanoma of The Cancer Genome Atlas data and meta-Gene Expression Omnibus cohorts.

## Abbreviations

TCGA_SKCM, cutaneous melanoma of TCGA data; GEO, Gene Expression Omnibus; ssGSEA, single-sample gene set enrichment analysis; CPI, clustering prediction index; TMB, mutation burden; FGA, fraction genome altered; AUC, area under the curve.
