# Supplementary figures and images for: Crosstalk of four kinds of cell deaths defines subtypes of cutaneous melanoma for precise immunotherapy and chemotherapy
Source: Front Immunol. 2022 Nov 30;13:998454. doi: 10.3389/fimmu.2022.998454 (PMC9747944; doi:10.3389/fimmu.2022.998454)

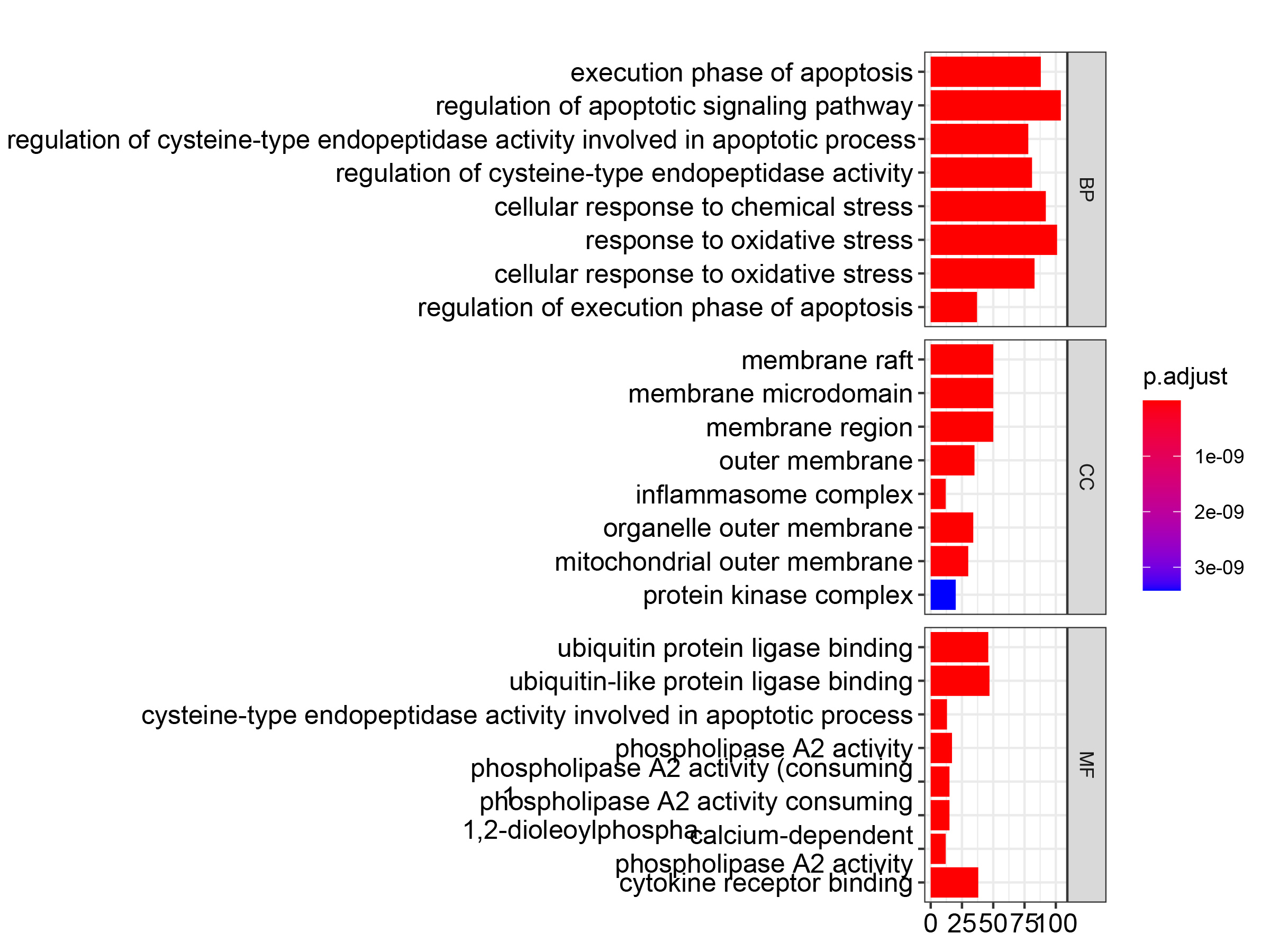

Supplement: Supplementary Figure 1 — The Gene Ontology enrichment of cell death- related genes. [file Image_1.jpeg]

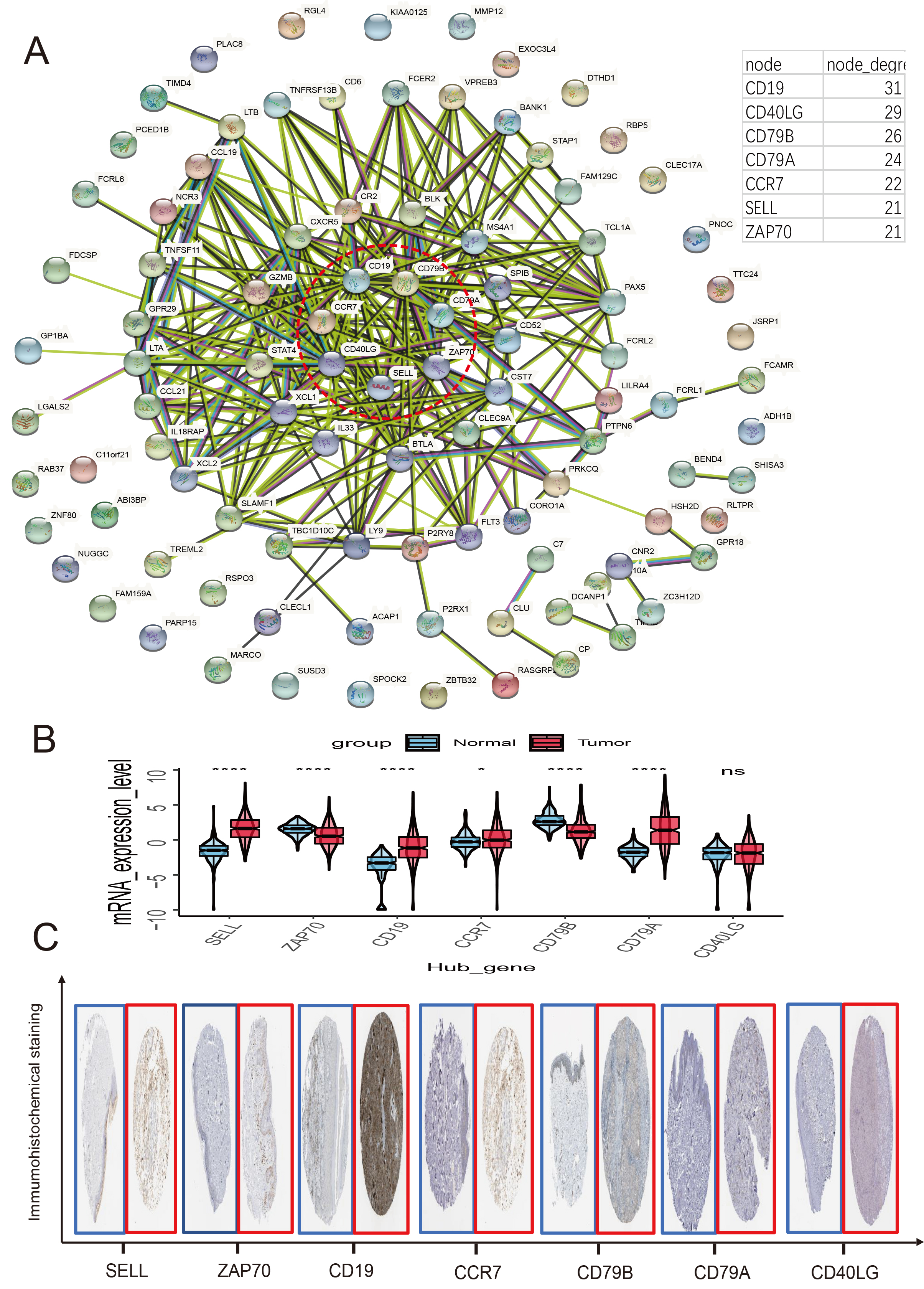

Supplement: Supplementary Figure 2 — Protein–protein interaction (PPI) network of CS4 subtype biomarkers. (A) The PPI network of biomarkers and seven hub genes. (B) Box plot of hub genes between tumor and normal skin groups in the Cancer Genome Atlas–Genotype Tissue Expression dataset. (C) Immunohistochemical images of hub genes in melanoma tissues and normal samples. [file Image_2.jpeg]
